# LOXL2 silencing suppresses angiotensin II-induced cardiac hypertrophy through the EMT process and TGF-β1/Smad3/NF-κB pathway 

**DOI:** 10.22038/IJBMS.2022.63338.13981

**Published:** 2022-08

**Authors:** Jun Luo, Yingbiao Wu, Xi Zhu, Saihua Wang, Xiaogang Zhang, Zhongping Ning

**Affiliations:** 1 Department of Cardiology, Shanghai University of Medicine & Health Sciences affiliated Zhoupu Hospital, Shanghai 201318, China; # These authors contributed eqully to this work

**Keywords:** Angiotensin II, Atrial fibrillation, Epithelial-mesenchymal – transition, Hypertrophy, LOXL2 protein

## Abstract

**Objective(s):**

Atrial fibrillation (AF) is a common arrhythmia with atrial myocyte hypertrophy linked with stroke, heart failure, and increased mortality. Lysyl oxidase-like 2 (LOXL2) involves the cross-linking of collagen in the extracellular matrix (ECM). In the present study, we investigated the roles and underlying mechanisms of LOXL2 on cardiomyocyte hypertrophy.

**Materials and Methods:**

The expression of LOXL2 mRNA and protein were detected in angiotensin II (Ang II) treated rat cardiomyocytes H9c2 by RT-qPCR and western blot. Small interfering RNA (siRNA) mediated LOXL2 gene silencing was used to evaluate cardiac hypertrophy and related markers. Also, the protein expression of EMT markers and Smad3/NF-κB pathway was determined by western blot.

**Results:**

Ang II significantly increased mRNA and protein expressions of LOXL2 and increased mRNA levels of myocardial hypertrophy markers, including ANP, BNP, and β-MHC in H9c2 cells. Silencing of LOXL2 significantly suppressed Ang II-induced hypertrophy and reversed the increase in ANP, BNP, and β-MHC mRNA levels. Also, EMT markers’ expressions, as evidenced by increased E-cadherin and decreased vimentin, α-smooth muscle actin (α-SMA), fibroblast-specific protein (FSP), and collagen 1A1. Mechanistically, we found that LOXL2 silencing suppressed protein expressions of TGF-β1, p-Smad3, and p-NF-κB in Ang II-stimulated H9c2 cells. LOXL2 silencing also attenuated Ang II-induced increased expression and content of proinflammatory cytokines IL-1β (H) and TNF-α.

**Conclusion:**

Our data speculated that LOXL2 might be a potential contributing factor to Ang II-induced cardiac hypertrophy, and TGF-β1/Smad3/NF-κB is involved in a signal axis and might be a potential strategy in treating cardiac hypertrophy.

## Introduction

Atrial fibrillation (AF) is a common arrhythmia with higher mortality and stroke risk than sinus rhythm (1). Atrial structural remodeling is a key pathological feature of AF, which plays a central role in maintaining arrhythmia (2). The fibrous tissue in the atria of AF patients is associated with AF development, progression, recurrence, the risk for complications, and therapeutic failure (3). With the development of diagnostic methods such as clinical fibrosis imaging, it is essential to detect cardiac fibrosis and hypertrophy to guide AF therapy (4). Therefore, it is urgently needed to explore the detailed molecular mechanisms underlying cardiac hypertrophy to provide new targets to develop therapeutic strategies for AF.

Cardiac hypertrophy is a compensatory response to the physical or pathological stimulus, leading to larger cardiomyocyte size. However, continuous hypertrophy will enter a decompensation period and results in heart failure and sudden death (5). Cardiac hypertrophy activates the transcription of several marker genes, such as atrial natriuretic peptide (ANP), brain natriuretic peptide (BNP), and β-myosin heavy chain (β-MHC) (6). The renin-angiotensin system (RAS) is an important signal that regulates cardiac hypertrophy and involves atrial structural remodeling and cardiac hypertrophy of AF (7). As a member of the RAS system, angiotensin II (Ang II) induces vasoconstriction by promoting vasopressin release from the central nervous system. Therefore, Ang II is a crucial stimulating agent for cardiovascular diseases with cardiac hypertrophy, such as hypertension, heart failure, and atrial fibrillation (8, 9).

Lysyl oxidase‐like 2 (LOXL2) belongs to the lysyl oxidase-like family, consisting of LOXL1–LOXL4 (10). LOXL2 catalyzes crosslinking of collagens and elastin (11), especially in the extracellular matrix (ECM) remodeling process (12). Therefore, it is also a potential contributor to cardiac hypertrophy. The gene expression of LOXL2 is increased in cardiac tissue of spontaneously hypertensive rats with left ventricular hypertrophy (13). Higher expression of LOXL2 has been found in hypoxia-exposed mice’s lungs, which developed pulmonary hypertension and showed increased collagen crosslinking right ventricular hypertrophy (14). The association of LOXL2 with cardiac hypertrophy is also confirmed in several cardiovascular diseases with cardiac hypertrophy. One study found an association between LOXL2 levels and cardiac fibrosis in heart failure (HF). LOXL2 is up-regulated in the cardiac interstitium in diseased human hearts and serum of HF patients, and the LOXL2 levels were correlated with collagen crosslinking and cardiac dysfunction (15). A recent study shows that serum LOXL2 levels were significantly elevated in AF patients compared with the healthy controls and correlated with left atrial fibrosis (16). Recent studies directed the pathogenic role of LOXL2 in cardiac fibrosis. However, the detailed mechanism of LOXL2 in cardiac hypertrophy remains unclear.

In this study, we aimed to explore LOXL2 as a potential contributor to cardiomyocyte hypertrophy developments. A hypertrophy cellular model was established in rat myocyte cell line H9c2 stimulated with Ang II. The expression of LOXL2 and myocardial hypertrophy marker will be evaluated. LOXL2 silencing effect on TGF-β1/Smad3/NF-κB pathway as well as the inflammatory response will be determined. Also, the myocardial hypertrophy marker and EMT marker will be evaluated in Ang II-induced H9c2 cells.

## Materials and Methods

***Cell culture***

The neonatal rat cardiomyocyte H9c2 cell line was purchased from the Chinese Academy of Sciences (Shanghai, China) and cultured in high glucose DMEM medium with 10 % FBS in a 5 % CO_2_ humidified incubator at 37 °C. When cell culture reached 70–80% confluence, it was treated with serum-free DMEM medium overnight. The cell culture was then treated with 1 μM Ang II for 48 hr (17).

***Cell transfection***

H9c2 cells were cultured in DMEM medium supplemented with 10% FBS. Cells were incubated at 37 °C with 5% CO2. Short interfering RNAs (siRNAs) targeting the LOXL2 gene (siLOXL2) were designed and synthesized (GenePharma, Shanghai, China). The nonsense siRNA was also synthesized to act as a negative control (siNC). The siRNA sequence targeting LOXL2 included: sense 5’-TGA TGG CTC ATG CCT GTA ATC-3’; antisense: 5’-GAT TAC AGG CAT GAG CCA TCA T-3’. The siRNA sequence for negative control was: sense 5’-TCT GTG ATT CTC GGT AAC ACG-3’, antisense 5’-CGA CAC ACG ATG CAT ATT GTG A-3’. When H9c2 cells were grown to 30–40% confluence, they were transfected with LOXL2 siRNA or NC (final concentration: 100 nM) using Lipofectamine 2000 (Life Technologies, NY, USA). After 48 hr, transfection efficiency was confirmed using Western blot and RT-qPCR analysis (18).

***Immunofluorescence analysis and cell surface area measurements***

H9c2 cells were fixed in 4 % paraformaldehyde for 20 min, permeabilized in 0.5 % Triton X-100 for 20 min, and blocked in 1 % bovine serum albumin (BSA) for 30 min at room temperature. Then cells were incubated overnight at 4 °C with primary antibodies to α-actinin (1:200, Alexa Fluor 488, Invitrogen). After washing with PBS, cells were mounted using a fluorescence quenching solution (Sigma-Aldrich, USA). The samples were stained with DAPI (1:1000, Sigma-Aldrich) to mark the nucleus. All images were detected using a fluorescence microscope (Olympus, BX60, Japan) and analyzed with ImageJ software. The surface area of H9c2 cells was calculated from at least 50 randomly chosen cells (19).

***Proinflammatory cytokine measurement by ELISA***

The culture media were collected from H9C2 cells, and ELISA was performed to determine IL-1β (cat.no. RLB00, R&D Systems, USA) and TNF-α (cat.no. RTA00, R&D Systems, USA) using ELISA kits. A microplate reader was applied to measure the absorbance at 450 nm. The IL-1β and TNF-α were calculated based on the standard curve and were expressed as pg/ml.

***Real-time quantitative PCR (RT-qPCR)***

Total RNA was extracted by Trizol (Invitrogen) and was reversely transcribed into cDNA using the Prime Script (R) RT reagent kit (TaKaRa Bio Inc, Dalian, China). Real-time PCR was performed in triplicate using the SYBR Premix Ex Taq kit (TaKaRa Bio Inc) on a RealTime PCR System (Applied Biosystems, Foster City, CA, USA). The primer sequences for LOXL2, ANP, BNP, IL-1β, TNF-α, and GAPDH are shown in Table 1. The PCR condition was set as follows: denaturing at 94 °C for 10 min, followed by 40 cycles at 94 °C for 15 sec and 58 °C for 30 sec. The data were normalized to GAPDH as an internal control, and RNA levels were analyzed with the 2−ΔΔCt method (20).

***Western blot***

Total proteins were extracted from H9c2 cells using RIPA extraction reagents (Solarbio, Beijing, China) and quantified by BCA Protein Assay Kit (Beyotime, Shanghai, China). Protein samples (50 μg) were separated by 10% SDS-PAGE and transferred onto PVDF membranes. The membranes were blocked with 5% BSA and incubated with the primary antibodies against LOXL2 (1:200; sc-293427; Santa Cruz, USA), E-cadherin (1:1000; sc-8426; Santa Cruz, USA), Vimentin (1:500; sc-32322; Santa Cruz, USA), α-SMA (1:500; sc-53142; Santa Cruz, USA), FSP (1:200; ab197896; Abcam, UK), Collagen 1A1 (1:1000; ab255809; Abcam, UK), TGF-β1 (1:500; ab179695; Abcam, UK), p-Smad3 (1:500; #9520; Cell Signaling), p-NF-κB (1:500; sc-166748; Santa Cruz, USA), and NF-κB (1:1000; sc-8008; Santa Cruz, USA) at 4 °C overnight. The membranes were then incubated with horseradish peroxidase-linked secondary antibody (1:1000) for 1 hr at room temperature. The proteins were detected with an ECL chemiluminescent detection system (Thermo Scientific, Waltham, MA, USA). β-actin was used as an internal control. The density of bands was analyzed using ImageJ software.

***Statistical analysis***

Data were presented with the mean ± standard error of the mean (SEM) and analyzed using SPSS 20.0 software (SPSS, Inc., Chicago, IL, USA). The differences among multiple groups were analyzed by one-way ANOVA, followed by the Bonferroni test. *P*<0.05 was considered statistically significant.

## Results

***LOXL2 expression was increased in Ang II-induced cardiomyocytes***

**Figure 1:**
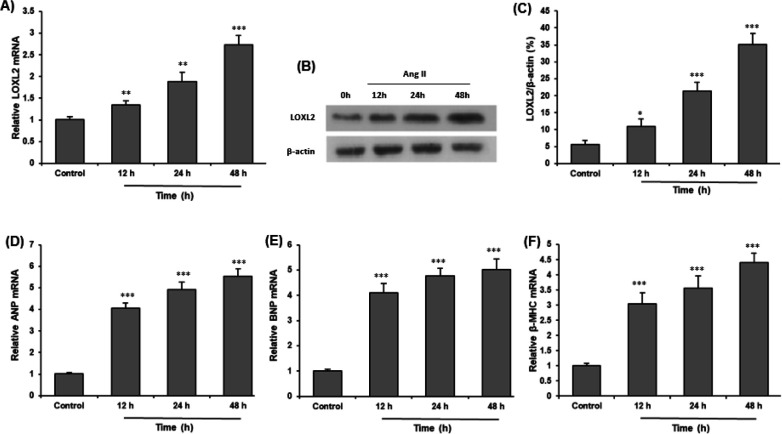
LOXL2 expression in Ang II-induced cardiomyocytes. Cultured H9c2 cells were treated by vehicle (Control) or Ang II (1 μM) for 12, 24, and 48 hr. A: mRNA expression level of LOXL2 in Ang II-induced H9c2 cells. B: Western blot was carried out to determine the protein expression level of LOXL2 in Ang II-induced H9c2 cells. C: Protein expression level of LOXL2 is increased by Ang II treatment in H9c2 cells. mRNA expressions of D: ANP, E: BNP, and F: β-MHC genes were evaluated by RT‐qPCR. All data are expressed as mean ± SEM, and the experiment was performed three times. *P<0.05, **P<0.01, and ***P<0.001 vs control group

H9c2 cells were treated with Ang II to determine the expression of LOXL2. Ang II treatment markedly increased the mRNA and protein expressions of LOXL2 in H9c2 cells in a time-dependent manner (*P*<0.05) (Figure 1 A-C). To explore the function of LOXL2 in cardiac hypertrophy, the mRNA expressions of cardiomyocyte hypertrophy markers including ANP, BNP, and β-MHC, were measured. We found that Ang II also increased mRNA levels of ANP, BNP, and β-MHC in cardiomyocytes in a time-dependent manner (*P*<0.05) (Figure 1D-F).

***LOXL2 silencing inhibited Ang II*** ***‐*** ***induced hypertrophic responses in cardiomyocytes***

**Figure 2:**
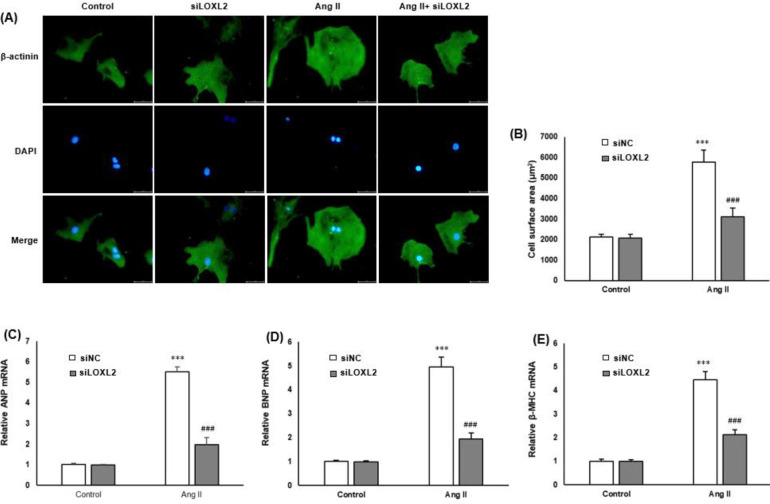
SiLOXL2 inhibits Ang II‐induced cardiomyocyte hypertrophy. H9c2 cells were transfected with siLOXL2 or siNC and treated with Ang II (1 μM) for 48 hr. A: Representative images of cardiomyocyte morphology by immunofluorescent staining with α‐actinin (green) and DAPI (blue) (scale bar: 5 μm). B: Surface area of cardiomyocytes is quantified by measuring 100 cardiomyocytes from each group. C: RT‐qPCR is performed to evaluate the mRNA expressions of ANP, D: BNP, and E: β-MHC genes; GAPDH is used as the internal control. ***P<0.001 vs cardiomyocytes in the control; ###*P*<0.001 vs cardiomyocytes with Ang II

To examine the role of LOXL2 in Ang-II induced cardiomyocyte hypertrophy, the LOXL2 expression was silenced using siRNAs specifically targeting LOXL2 in cardiomyocytes. Immunofluorescence was carried out in H9c2 cells with an antibody of α-actinin to explore the function of LOXL2 silencing in cardiac hypertrophy. Ang II significantly increased the hypertrophy of H9c2 cells, as evidenced by increased cardiomyocyte size (cell surface area). However, H9c2 cells transfected with siLOXL2 had a suppressive hypertrophic response to Ang II, with significantly reduced cardiomyocyte size (Figure 2A-B). The mRNA expressions of cardiomyocyte hypertrophy markers, including ANP, BNP, and β-MHC, were decreased in Ang II-treated siLOXL2 cardiomyocytes (Figure 2 C-E).

***LOXL2 silencing suppressed the EMT process in H9c2 cells***

**Figure 3:**
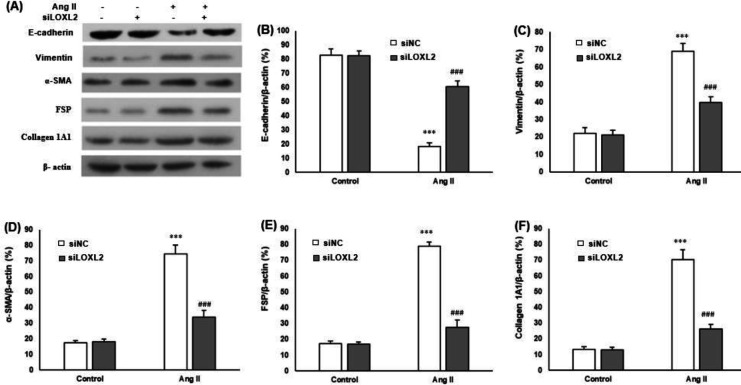
Effects of siLOXL2 on EMT markers in cardiomyocytes. Cultured H9c2 cells were transfected with siLOXL2 or siNC, and treated with Ang II (1 μM) for 48 hr. A: Western blot is performed to assess protein levels of EMT markers, and representative bands are shown. Bands are quantified by ImageJ software and show that siLOXL2 significantly attenuates the decrease in B: E-cadherin, and attenuates the increases in C: Vimentin, D: α-SMA, E: FSP, and F: Collagen 1A1 protein expressions. β-actin served as a loading control. ***P<0.001 vs cardiomyocytes in the control; ###*P*<0.001 vs cardiomyocytes with Ang II

To observe whether LOXL2 influences the Ang II-induced EMT process of cardiomyocytes, H9c2 cells were transiently transfected with siLOXL2 or siNC and incubated with Ang II (1 μM) for 48 hr. Western blot was performed to determine the protein expressions of EMT markers (Figure 3A). After treatment with Ang II for 48 hr, the expression of E-cadherin protein was significantly suppressed (Figure 3B). In contrast, the expressions of Vimentin (Figure 3C), α-SMA (Figure 3D), FSP (Figure 3E), and collagen 1A1 (Figure 3F) proteins were significantly increased (all *P*<0.05). However, these changes induced by Ang II were reversely by siLOXL2.

***LOXL2 silencing inhibited TGF-β1/Smad3/NF-κB pathway in cardiomyocytes induced by Ang II***

**Figure 4:**
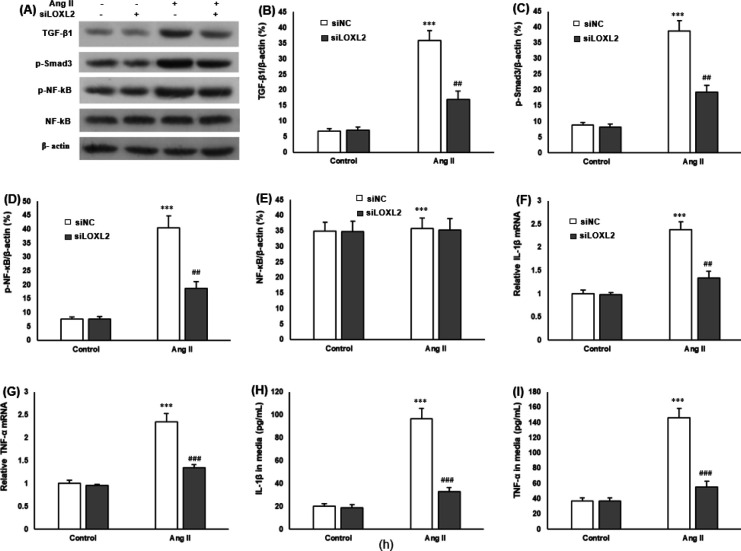
Effect of siLOXL2 on the expression of TGF-β1/Smad3/NF-κB pathway and inflammatory response. H9c2 cells are transfected with siLOXL2 or siNC and incubated with Ang II for 48 hr. A: Western blot was performed and shows the representative bands. siTNC significantly attenuates the Ang II-induced increase in B: TGF-β1, C: p-Smad3, and D: p-NF-κB, but does not change the E: NF-κB protein level. β-actin served as a loading control. RT-qPCR is performed to detect the mRNA levels of F: IL-1β and G: TNF-α in H9c2 cells. ELISA was performed to detect the secreted H: IL-1β and I: TNF-α in the cell supernatant of H9c2 cells. ***P<0.001 vs cardiomyocytes in the control; ##*P*<0.01 and ###*P*<0.001 vs cardiomyocytes with Ang II

Western blot was performed to explore whether LOXL2 silencing affects the TGF-β1/Smad3/NF-κB pathway (Figure 4A). These results showed that Ang II increased the expression of TGF-β1 (Figure 4B), p-Smad3 (Figure 4C), and p-NF-κB (Figure 4D) in H9c2 cells, which was markedly attenuated by LOXL2 silencing (*P*<0.05). The expression of total NF-κB in H9c2 cells remained unchanged (*P*<0.05) (Figure 4E).

***LOXL2 silencing inhibited the inflammatory response of cardiomyocytes induced by Ang II***

RT-qPCR and ELISA determined the expression and production of inflammatory cytokines. Ang II significantly enhanced mRNA expressions of IL-1β and TNF-α in H9c2 cells, and these changes were both reversed by LOXL2 silencing (Figure 4 F-G). Similar results were found in supernatant inflammatory cytokines. Compared with the Ang II group, IL-1β and TNF-α secretion in culture media decreased (*P*<0.05) (Figure 4 H-I).

## Discussion

In this study, we explored the effect of LOXL2 in H9c2 cells stimulated with Ang II. The principal findings were that Ang II increased LOXL2 expression in H9c2 cells. LOXL2 silencing by siRNA markedly suppressed cardiac hypertrophy and proinflammatory cytokine production. LOXL2 silencing suppressed the EMT process, with increased E-cadherin and decreased Vimentin expression, α-SMA, FSP, and Collagen 1A1 markers. LOXL2 silencing also inhibited protein expressions of TGF-β1 and phosphorylation of Smad3 and NF-κB in Ang II-stimulated H9c2 cells. Our findings showed that LOXL2 silencing has protective effects by inhibiting cardiac hypertrophy and inflammation in cardiomyocytes induced by Ang II. The mechanism may be related to the inhibition of the EMT process TGF-β1/Smad3/NF-κB signaling pathway.

LOXL2 catalyzes crosslinking of collagens and elastin and is a contributor to cardiac hypertrophy. Therefore, LOXL2 expression is increased in diseases with cardiac hypertrophy, such as cardiac tissue of hypertensive rats with left ventricular hypertrophy (13) and lung tissue of hypoxia-exposed pulmonary hypertension mice (14). Moreover, serum LOXL2 levels in AF patients were significantly higher than those of the healthy controls and correlated with left atrial fibrosis (16). Therefore, we hypothesized that LOXL2 mediates hypertrophy of Ang II-induced cardiomyocytes. Our experiment showed increased mRNA and protein of LOXL2 after Ang II treatment. Silencing LOXL2 expression by siRNA attenuated the increase in cardiomyocyte size and mRNA of hypertrophic markers, ANP, BNP, and β-MHC. The results indicate LOXL2 might involve the process of cardiac hypertrophy. Our results are by another report that LOXL2 inhibition by lysyl oxidase inhibitor, β-aminopropionitrile, attenuated right ventricular hypertrophy, and normalized collagen crosslinking in hypoxia-exposed pulmonary hypertension mice (14). Cardiac hypertrophy could be promoted by several members of the lysyl oxidase family. Transgenic overexpression of LOX or LOXL1 aggravated cardiac hypertrophy in mice with or without angiotensin II induction (21, 22). Our results add LOXL2 as a new hypertrophy inducer of the lysyl oxidase family. 

Previous studies show that LOXL2 is elevated in the serum of heart failure or atrial fibrillation patients (15, 16). This serum LOXL2 is likely secreted from fibroblasts in the cardiac interstitium, supported by the high expression of LOXL2 in the cardiac interstitium of heart failure patients (15). However, the expression of LOXL2 in cardiomyocytes is low, and whether LOXL2 has a hypertrophic role in cardiomyocytes remains unclear in HF and AF patients. This study shows that LOXL2 expression can be induced by Ang II treatment in H9c2 cardiomyocytes. Ang II also enhanced the EMT process, increased EMT marker Vimentin and α-SMA, and collagen gene FSP and collagen 1A1. Epithelial-mesenchymal transition (EMT) is a highly regulated pathological process and plays a regulatory role in cardiac development, repair, and fibrosis (23).

Moreover, during fibrogenesis, EMT directly leads to converting fibroblast into myofibroblast, a type of cell that could produce collagen. Therefore, EMT promotes the development and progression of cardiac fibrosis and hypertrophy (24, 25). The following experimental results can support the role of EMT in LOXL2-induced cardiac fibrosis. In cardiac stress mice, LOXL2 was expressed in fibroblasts of the interstitium, accompanied by transdifferentiation of fibroblasts into myofibroblasts, with enhanced expression of α-SMA and collagen 1A1 (15). It seems that LOXL2 is not expressed in physical cardiomyocytes but expressed in fibroblasts and myofibroblasts that transformed from cardiomyocytes through EMT, as in our Ang II-treated H9c2 cells. LOXL2 is essential for the EMT process in various tumor cells (26, 27). This is the first report about the modulation of LOXL2 on EMT in cardiac hypertrophy. Taken together, EMT mediates the inhibition of LOXL2 silencing on Ang II-induced cardiomyocyte hypertrophy.

Our results showed that Ang II-induced protein expressions of TGF-β1, p-Smad3, and p-NF-κB in cardiomyocytes were markedly reversed by LOXL2 silencing. TGF-β1 is a cytokine that plays a critical role in EMT progression (28). TGF-β1-induced EMT plays a vital role in cardiac fibrosis and hypertrophy (29, 30). Smad3 is a downstream protein of TGF-β1 and constitutes a TGF-β1/Smad3 axis, a potential target in combating cardiac hypertrophy. Inhibition of the TGF-β1/Smad3 axis suppressed cardiac hypertrophy and suppressed fibroblast-to-myofibroblast transformation (31). TGF-β1/Smad3 axis also stimulated CTGF expression and caused collagen type I generation in fibroblasts (32). LOXL2 lies upstream of Smad3 in that silencing LOXL2 inhibited the expression of pSmad3 in lung fibroblasts of bleomycin-induced pulmonary fibrosis mice (33). Moreover, LOXL2 knockdown reversed TGF-β-mediated induction of endothelial-to-mesenchymal transition (EndMT) in endothelial cells (34). However, there are controversial results in the regulation between TGF-β1 and LOXL2. Stimulation with TGF-β1 up-regulated LOXL2 expression in human chondrocytes (35), while the TGF-β1/Smad3 pathway lies downstream of LOXL2 in trophoblast cells (36). Our study shows reduced TGF-β1 and p-Smad3 proteins in H9c2 cells with LOXL2 silencing and indicate modulation of the TGF-β1/Smad3 axis by LOXL2. As overexpression of LOXL2 alone is insufficient to induce EndMT in endothelial cells (34), the possibility cannot be ruled out that TGF-β1 enhances LOXL2 expression making a positive feedback loop in cardiac hypertrophy.

**Table 1:** List of oligonucleotide primer sequences used in this study

**Gene**	**Primer name**	**Sequence (5'----3')**
LOXL2	LOXL2-F	GCATGGATTTGGCATGACTG
	LOXL2-R	GCACACTCGTAACTCTTCTG
ANP	ANP-F	ACCAAGGGCTTCTTCCTCT
	ANP-R	TTCTACCGGCATCTTCTCC
BNP	BNP-F	AGAACAATCCACGATGCAGAAG
	BNP-R	AAACAACCTCAGCCCGTCACA
β-MHC	β-MHC-F	AAGGGCCTGAATGAGGAGTA
	β-MHC-R	AAAGGCTCCAGGTCTGAGG
IL-1β	IL-1β-F	GCTGTGGCAGCTACCTATGTCTTG
	IL-1β-R	AGGTCGTCATCATCCCACGAC
TNF-α	TNF-α-F	GATCGGTCCCAACAAGGAGG
	TNF-α-R	GCTTGGTGGTTTGCTACGAC
GAPDH	GAPDH-F	CCTCTATGCCAACACAGTGC
	GAPDH-R	GTACTCCTGCTTGCTGATCC

LOXL2: Lysyl oxidase-like 2; ANP: Atrial natriuretic peptide; BNP,: Brain natriuretic peptide; β-MHC: β-Myosin heavy chain; GAPDH: Glyceraldehyde-3-phosphate dehydrogenase

## Conclusion

Ang II increases mRNA and protein expressions of LOXL2 and promotes cardiac hypertrophy and inflammatory cytokine production. Meanwhile, LOXL2 silencing protects against Ang II-induced cardiac hypertrophy via inhibition of the EMT process and down-regulation of the TGF-β1/Smad3/NF-κB pathway. Therefore, our results suggest LOXL2 as a contributing factor for cardiac hypertrophy, thus providing LOXL2 as a therapeutic target for cardiovascular diseases with cardiac hypertrophy and deregulated TGF-β1/Smad3 pathway, including atrial fibrillation.

## Authors’ Contributions

JL Performed experiments and wrote the manuscript; YW Performed experiments and revised the manuscript; XZ and SW Performed experiments; XZ Provided statistical analysis; ZN Conceived the idea, designed and supervised the study.

## Conflicts of Interest

The authors declare no conflicts of interest with other people or organizations.
